# Morphometric Analysis of the Incisive (Nasopalatine) Canal and Foramen: Clinical Implications for Anterior Maxillary Surgery

**DOI:** 10.7759/cureus.89573

**Published:** 2025-08-07

**Authors:** Ijaz Ur Rehman, Malik Adeel Anwar, Irum Farheen, Salman Amin, Rehma Sammar, Tahmasub Faraz Tayyab, Hooria Kushef, Arooj Naeem, Moghees Ahmed Baig

**Affiliations:** 1 Department of Oral Medicine and Radiology, University College of Medicine and Dentistry, The University of Lahore, Lahore, PAK; 2 Department of Biomedical Engineering, Binghamton University, State University of New York, Binghamton, USA; 3 Department of Oral Pathology and Oral Diagnostics, University College of Medicine and Dentistry, The University of Lahore, Lahore, PAK; 4 Department of Oral and Maxillofacial Surgery, University College of Medicine and Dentistry, The University of Lahore, Lahore, PAK; 5 Department of Oral and Maxillofacial Surgery, Niazi Medical and Dental College, Sargodha, PAK; 6 Department of Medicine, Faisalabad Medical University, Faisalabad, PAK; 7 Department of Dentistry, University College of Medicine and Dentistry, The University of Lahore, Lahore, PAK

**Keywords:** anterior maxilla, cone beam computed tomography (cbct), dental implants, incisive canal, incisive foramen, nasopalatine canal, nasopalatine foramen

## Abstract

Background and aim: The incisive (nasopalatine) canal is an important anatomical structure of the anterior maxilla. It holds significance for surgeries and implant placement in the central incisor region. The size, shape, and relation with surrounding bones may vary by age, gender, and ethnicity. Standard radiographs used in dentistry often miss these variations, while cone beam computed tomography (CBCT) allows a precise evaluation in sagittal and axial views. This study aimed to analyze anatomical variations of the incisive canal and surrounding structures, such as canal length, shape, diameter, and bone thickness in the Pakistani subpopulation using CBCT, and compare them across age and gender.

Methods: A retrospective cross-sectional study was conducted on 121 high-resolution CBCT scans taken from the Oral Radiology Department, the University of Lahore, from 2021 to 2023. Measurements were done in Planmeca Romexis software (Helsinki, Finland: Planmeca Oy), and statistical analysis was performed using SPSS version 25 (Armonk, NY: IBM Corp.) using t-tests, ANOVA, and nonparametric tests where appropriate.

Results: The mean age was 34.61±15.38 years. Males had significantly longer incisive canals, thicker palatal bone, and longer incisor roots (p<0.05). Posterior bone width and the distance from incisive foramen to alveolar crest were also greater in males, the latter being marginally significant. Anterior bone width decreased considerably in older age groups, especially above 60 years (p=0.010), while incisor root width showed no gender difference. Hourglass was the most common canal shape (43%), and round foramen shape was slightly more common than oval. No significant differences in shape were seen by age groups and gender.

Conclusion: This study shows key anatomical differences in the Pakistani subpopulation. Gender and age affected many linear measurements, while canal and foramen shapes were fairly consistent. The hourglass configuration was the predominant shape of the incisive canal, and the round foramen appeared slightly more frequently than the oval type. These research findings will be useful for implant planning and surgical procedures involving the anterior maxilla.

## Introduction

The maxillary incisive canal, also called the nasopalatine canal, situated in the front part of the maxilla, has a vital function in supplying sensory nerves to the nasal septum and hard palate [1]. The canal can have many shapes, such as single, double, or triple canals. In certain situations, irregular septa can be seen [2]. The position of the maxillary central incisors in relation to the incisive canal is affected by sagittal skeletal malocclusions, resulting in varying distances observed in class I, II, and III malocclusions [3]. The proximity of certain anatomical structures, such as the palatal plane, maxillary alveolar border, incisive canal, and maxillary central incisors, can influence the results of operations that involve moving the front teeth backwards [4]. Comprehending these anatomical and morphological elements is essential for planning and performing surgical procedures, administering local anesthetics, and placing dental implants specifically in the anterior part of the maxilla [5].

Placing implants immediately after extraction is quite common in today's dental practice. However, performing this procedure in the region of the maxillary central incisor can be challenging due to the presence of complex and closely associated anatomical structures [4]. The nasopalatine (incisive) canal, which opens into the oral cavity through the incisive foramen and connects to the nasal cavity via the nasopalatine (incisive) foramen, contains important nerves and blood vessels such as the nasopalatine nerve and a vascular anastomosis between the greater palatine and sphenopalatine arteries. If an implant accidentally enters the canal, it may cause nerve damage, an altered sensation in the area, or poor integration with the bone. Research has shown that canals can vary not just in size but also in shape and position relative to the surrounding structures. It can be anywhere between cylindrical, funnel-shaped, banana-shaped, or hourglass-shaped. In some patients, the canal is wide and the bone in front of it is very thin, which makes the implant placement risky or even not possible [6]. There is a concern that during such procedures the implant might unintentionally breach into the nasal cavity or the canal itself, leading to complications during or after implant surgery. Such contact between the implant and neural tissue can lead to challenges in achieving osseointegration, intraoperative bleeding, and postoperative sensory impairments [3].

Traditionally, standard two-dimensional radiographs have been the go-to method for diagnosing and planning treatments. However, they fall short when it comes to accurately assessing structures like the incisive canal, cortical plate, and alveolar bone housing on the premaxilla [5]. Recent advancements in radiographic technology offer enhanced precision and detail in assessing oral conditions. Among these technologies, cone beam computerized tomography (CBCT) stands out as a highly accurate method for three-dimensional imaging of hard tissue, with minimal distortion and reduced radiation exposure [7,8]. CBCT enables comprehensive visualization of axial, coronal, and sagittal planes, particularly beneficial for evaluating the sagittal plane of the incisive canal. This imaging modality allows for detailed examination of the canal's shape, curvature, angle, and length, offering valuable insights into anatomical variations as described above [6,9].

While intraoral radiographs also offer some insight into incisive canal anatomy, CBCT provides superior resolution and clarity, particularly for detecting variations in canal shape and position [10,11]. Therefore, for a comprehensive evaluation of incisive canal anatomy, particularly on sagittal sections, CBCT emerges as the optimal imaging technique, providing high-resolution images essential for clinical assessment.

The objective of the study was to assess anatomical variations of the incisive canal and surrounding structures - including canal length, diameter, shape, and its relation to adjacent bone in sagittal and axial views - across different age groups and genders, with emphasis on implications for the diagnosis, treatment planning, and implementation of maxillary implant surgery.

## Materials and methods

The retrospective observational descriptive study utilized purposive sampling of CBCT data from 121 cases obtained from the Department of Oral Radiology, University College of Dentistry, The University of Lahore, spanning between 2021 and 2023. Ethical clearance was obtained from the University College of Dentistry, The University of Lahore's Institutional Review Board, certified under #UCD/ERCA/24/181.

The CBCT imaging was performed using a Planmeca ProMax unit under standardized conditions as follows: 5.6 mA, 90 kV, and a voxel size of 400 µm and a 12 s scan time. Image dimensions were 23.0x27.6 cm (576x576x689 voxels). All measurements were performed using Planmeca Romexis software.

A total of 160 CBCT scans were initially reviewed, and after applying exclusion criteria, 121 diagnostic quality scans were selected through purposive sampling for final analysis. The inclusion criteria included patients of both genders, aged 10 years and above. Exclusion criteria involved individuals with missing maxillary incisors, those exhibiting apparent, missing or filled maxillary anterior teeth, nasopalatine pathology, periodontal disease, or congenital abnormalities, such as cleft lip and palate, and images of inadequate quality. These exclusions aimed to ensure a standardized and representative sample for analysis. Initially, CT scans of 160 patients were assessed, of which 39 were excluded.

**Figure 1:**
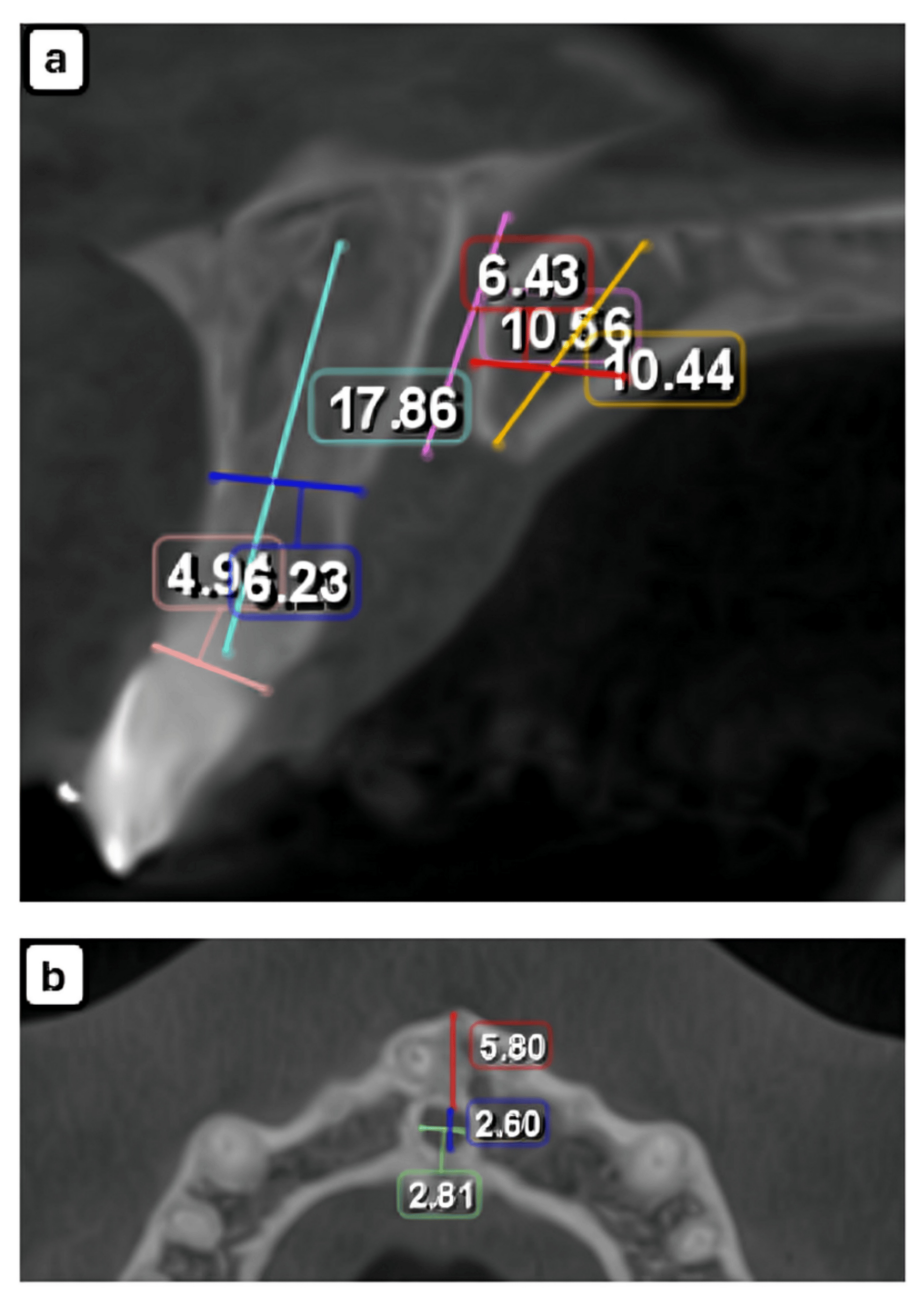
Anatomical parameters assessed on CBCT. Measurements performed on sagittal view (a) and axial view (b). Sagittal view: purple represents the length of the incisive canal. Yellow indicates the palatal bone length. Red marks the width of bone posterior to the canal. Blue denotes the width of bone anterior to the canal. Light blue shows the incisor root length measured from the cementoenamel junction to the apex. Pink refers to the incisor root width at the cementoenamel junction. Axial view: green represents the mesiodistal diameter of the incisive foramen. Blue indicates the labiopalatal diameter of the incisive foramen. Red shows the distance from the incisive foramen to the alveolar crest.

The study conducted a comprehensive anatomical assessment with clinical relevance. Measurements were categorized based on sagittal and axial CBCT views. In the sagittal view, the assessed parameters included the incisive canal length and diameter, incisor root length, buccal bone plate thickness at the canal level, and palatal bone length (Figure 1a). In the axial view, the diameter of the incisive canal (mesiodistal and labiopalatal) and the distance from the incisive foramen to the alveolar crest were measured (Figure 1b).

All data were entered and analyzed using SPSS version 25 (Armonk, NY: IBM Corp.). Quantitative variables, such as age, incisive canal length, incisor root length, and various bone dimensions, were expressed as mean with standard deviation. Qualitative variables like gender and canal shape were reported as frequencies and percentages. To analyze the data, both parametric and nonparametric tests were employed, depending on the distribution of the variables. The Kolmogorov-Smirnov test was used to assess the normality of the continuous variables. For comparing gender-based differences, the independent samples t-test was applied for normally distributed data, while the Mann-Whitney U test was used when the assumption of normality was not met. To compare more than two groups, such as age ranges, one-way ANOVA was performed for parametric data, and the Kruskal-Wallis H test was performed for nonparametric counterparts. Chi-square tests were applied to examine associations between categorical variables like canal shape and gender or age groups in the study. A p<0.05 was considered statistically significant.

## Results

The demographic data showed that the mean age of study participants was 34.61±15.38 years (range: 10-70 years). In this study, 56 (46.3%) participants were male and 65 (53.7%) were female. The mean age of male participants was 37.1±15.68 with an age range of 12-70 years, while for females the mean age was 32.5±14.91 with an age range of 10-69 years. Table 1 shows the descriptive characteristics of the various parameters for the participants.

**Table 1:** Descriptive statistics of sagittal and axial dimensions of anterior maxilla (n=121). CEJ: cementoenamel junction

Variables	Mean±SD (mm)	Min-max (mm)
Sagittal view		
Length of the incisive canal	10.47±2.42	5.73-16.88
Palatal bone length	10.80±2.21	6.32-16.92
Width of the bone anterior to the canal	6.43±1.59	2.51-10.01
Width of the bone posterior to the canal	3.56±1.02	1.60-7.07
Incisor root length from CEJ to apex	12.35±1.58	6.51-16.12
Incisor root width at CEJ	6.34±0.74	2.58-9.36
Axial view		
Diameter of incisive foramen (mesiodistal)	3.18±1.07	1.40-6.81
Diameter of incisive foramen (labiopalatal)	2.93±0.88	1.20-6.41
Distance of incisive foramen from the alveolar crest	6.74±1.46	3.80-11.40

Gender-based variations

Gender wise comparison revealed several statistically significant anatomical differences. Males consistently showed greater measurements in sagittal view parameters. Specifically, the length of the incisive canal, palatal bone length, and width of bone posterior to the canal were all significantly higher in males compared to females (p<0.05). The incisor root length from CEJ to apex was also significantly longer in males (12.98 mm vs. 11.80 mm, p<0.05). In contrast, no significant differences were observed between males and females in root width at CEJ or in the most axial view parameters measured. However, one axial parameter - the distance from the incisive foramen to the alveolar crest - was marginally significant in males (p=0.043) (Table 2).

**Table 2:** Comparison of sagittal and axial view dimension in relation to gender. ^a^Independent sample t-test. ^b^Mann-Whitney U test. CEJ: cementoenamel junction

View	Dimensions	Male (mm)		Female (mm)		p-Value	Test statistic
		Mean	SD	Mean	SD		
Sagittal	Length of the incisive canal	11.67	2.24	9.43	2.07	<0.05^a^	5.714
	Palatal bone length	11.96	2.31	9.80	1.54	<0.05^a^	6.139
	Width of the bone anterior to the canal	6.58	1.35	6.31	1.78	0.357^a^	0.924
	Width of the bone posterior to the canal	3.94	1.13	3.24	0.79	<0.05^b^	1119.5
	Incisor root length from CEJ to apex	12.98	1.37	11.80	1.56	<0.05^a^	4.382
	Incisor root width at CEJ	6.33	0.96	6.35	0.51	0.811^b^	1774
Axial	Diameter of incisive foramen (mesiodistal)	3.22	0.94	3.15	1.17	0.391^b^	1655
	Diameter of incisive foramen (labiopalatal)	3.04	0.93	2.84	0.83	0.242^b^	1595
	Distance of incisive foramen with the alveolar crest	6.97	1.25	6.55	1.60	0.043^b^	1430.5

Age-based variations

Age group analysis showed a significant difference in the width of the bone anterior to the canal, which showed a decreasing trend with increasing age. Participants aged 61-70 years had the narrowest anterior bone width (mean=4.85 mm), while those in the age group of 10-20 years had the widest (mean=6.94 mm) with a statistically significant p-value of 0.010. No other sagittal or axial parameters showed significant variation across age groups, though some minor fluctuations were seen (Table 3).

**Table 3:** Comparison of sagittal and axial dimensions across age groups. ^a^One-way ANOVA. ^b^Kruskal-Wallis H test. CEJ: cementoenamel junction

View	Dimensions	Age groups						p-Value	Test statistic
		10-20 (years)	21-30 (years)	31-40 (years)	41-50 (years)	51-60 (years)	61-70 (years)		
		Mean±SD (mm)	Mean±SD (mm)	Mean±SD (mm)	Mean±SD (mm)	Mean±SD (mm)	Mean±SD (mm)		
Sagittal	Length of the incisive canal	9.82±2.46	10.60±2.20	10.58±2.58	9.55±1.49	10.95±3.34	11.40±1.93	0.390^a^	1.053
	Palatal bone length	10.52±2.43	10.69±1.89	10.69±1.88	10.38±1.90	11.43±3.38	11.68±1.59	0.580^a^	0.760
	Width of the bone anterior to the canal	6.94±1.50	6.81±1.55	6.00±1.64	6.41±1.38	6.48±1.30	4.85±1.83	0.010^a^	3.167
	Width of the bone posterior to the canal	3.55±1.03	3.53±0.81	3.88±1.31	3.32±1.18	3.43±0.91	3.60±0.85	0.802^b^	0.441
	Incisor root length from CEJ to apex	11.83±1.82	12.02±1.49	12.63±1.23	12.64±1.61	12.81±1.38	12.73±2.26	0.226^a^	1.409
	Incisor root width at CEJ	6.43±0.69	6.44±0.97	6.32±0.57	6.43±0.67	6.05±0.57	6.16±0.60	0.674^b^	0.790
Axial	Diameter of incisive foramen (mesiodistal)	2.97±1.17	3.26±1.12	3.08±0.71	3.09±1.24	3.30±1.13	3.51±1.15	0.501^b^	1.382
	Diameter of incisive foramen (labiopalatal)	2.61±0.79	3.06±0.95	2.93±0.61	2.93±1.07	2.88±0.94	3.17±0.88	0.213^b^	3.089
	Distance of incisive foramen from the alveolar crest	6.94±1.24	7.02±1.43	6.33±1.61	6.93±1.07	6.92±1.43	5.58±1.35	0.217^a^	3.060

Shape of the incisive canal

**Figure 2:**
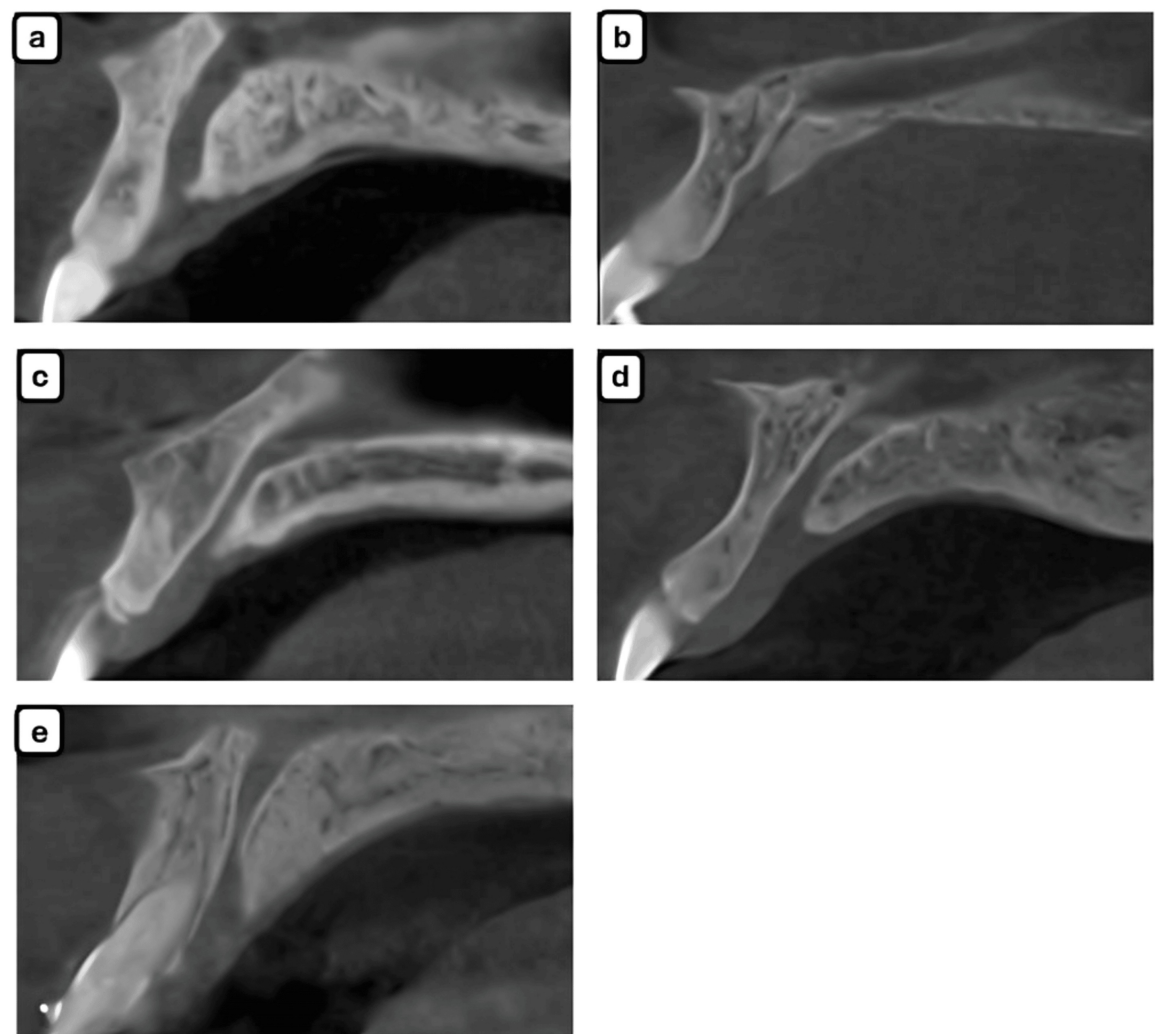
Shape of incisive canal. The following shapes have been observed: (a) banana, (b) cone, (c) cylindrical, (d) funnel, (e) hourglass.

As presented in Tables 4, 5, the hourglass shape was the most common shape of the incisive canal. The least observed shapes were cone and funnel-shaped. Distribution patterns did not significantly vary by gender or age group, though hourglass configuration remained consistently predominant across subgroups (Figures 2a-2e).

**Table 4:** Shape of incisive canal in relation to age. *Chi-square test.

Variables	Age groups												Total	p-Value*	Test statistic
	10-20 (years)		21-30 (years)		31-40 (years)		41-50 (years)		51-60 (years)		61-70 (years)				
	n	%	n	%	n	%	n	%	n	%	n	%			
Banana	5	26.3	9	24.3	4	17.4	6	37.5	8	47.1	3	33.3	35	0.611	17.634
Cone	0	0	2	5.4	1	4.3	0	0	1	5.9	1	11.1	5		
Cylindrical	7	36.8	7	18.9	6	26.1	2	12.5	1	5.9	1	11.1	24		
Funnel	2	10.5	2	5.4	1	4.3	0	0	0	0	0	0	5		
Hourglass	5	26.3	17	45.9	11	47.8	8	50.0	7	41.2	4	44.4	52		
Total	19	100.0	37	100.0	23	100.0	16	100.0	17	100.0	9	100.0	121		

**Table 5:** Shape of incisive canal in relation to gender. *Chi-square test.

Variables	Gender					p-Value*	Test statistic
	Male		Female		Total		
	n	%	n	%			
Banana	15	26.8	20	30.8	35	0.430	3.825
Cone	2	3.6	3	4.6	5		
Cylindrical	8	14.3	16	24.6	24		
Funnel	2	3.6	3	4.6	5		
Hourglass	29	51.8	23	35.4	52		
Total	56	100.0	65	100.0	121		

**Figure 3:**
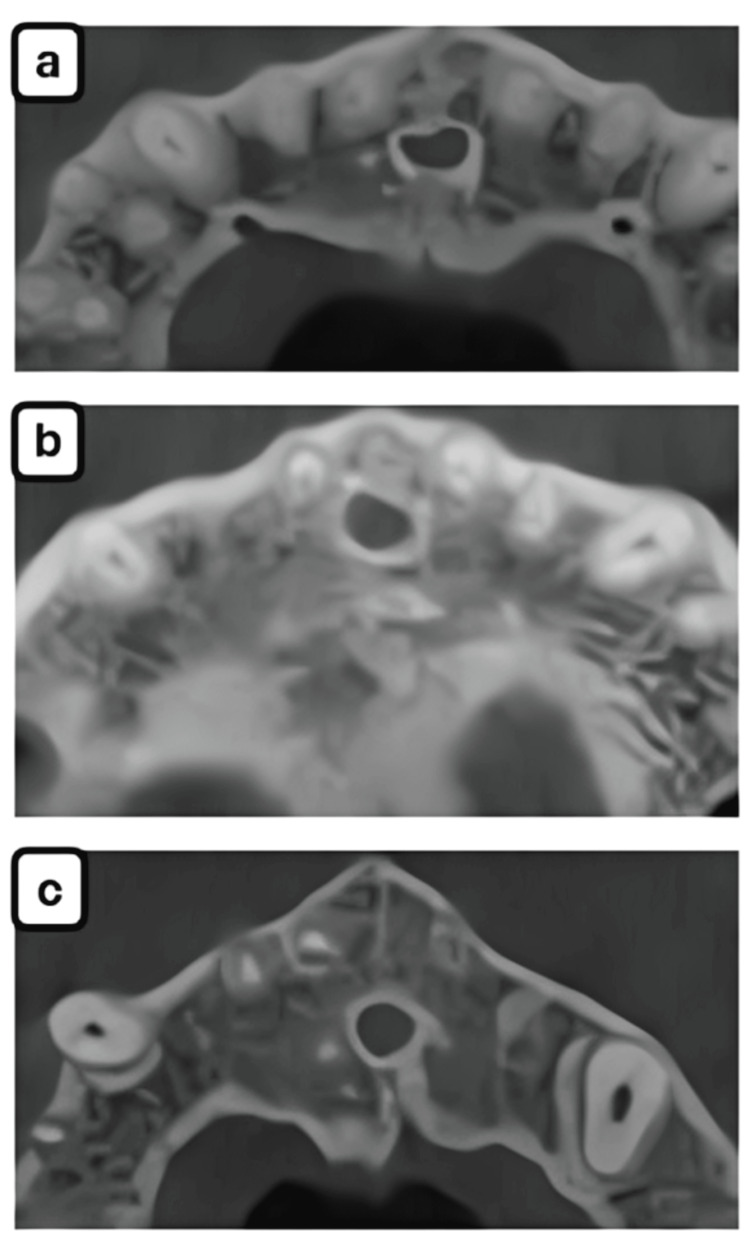
Shape of incisive foramen. The following shapes have been observed: (a) heart, (b) oval, (c) round.

In terms of incisive foramen morphology, the round shape was frequently observed (n=55, 45%), followed by oval and heart-shaped foramina. These trends were uniform across both gender and age groups, and no statistically significant differences were noted (Tables 6, 7) (Figures 3a-3c).

**Table 6:** Shape of incisive foramen in relation age. ^*^Chi-square test.

Variables	Age groups (years)												Total	p-Value*	Test statistic
	10-20		21-30		31-40		41-50		51-60		61-70				
	n	%	n	%	n	%	n	%	n	%	n	%			
Heart	3	15.8	7	18.9	3	13.0	3	18.8	1	5.9	1	11.1	18	0.542	8.894
Oval	7	36.8	16	43.2	11	47.8	7	43.8	3	17.6	4	44.4	48		
Round	9	47.4	14	37.8	9	39.1	6	37.5	13	76.5	4	44.4	55		
Total	19	100.0	37	100.0	23	100.0	16	100.0	17	100.0	9	100.0	121		

**Table 7:** Shape of incisive foramen in relation to gender. ^*^Chi-square test.

Variables	Gender				Total	p-Value*	Test statistic
	Male		Female				
	n	%	n	%			
Heart	9	16.1	9	13.8	18	0.884	2.46
Oval	21	37.5	27	41.5	48		
Round	26	46.4	29	44.6	55		
Total	56	100.0	65	100.0	121		

## Discussion

This CBCT-based study aimed to analyze the morphometric differences of the maxillary incisive canal and foramen across different age groups and between genders, with emphasis on their clinical relevance in surgical procedures involving the anterior maxilla. With 121 participants, this study contributes to region-specific anatomical data and highlights several statistically and clinically significant trends, some of which align with previous studies, while others don’t, suggesting potential ethnic variation.

The mean length of the incisive canal in this study was 10.47±2.42 mm, with males demonstrating longer canals than females. This is consistent with findings by Soumya et al., who reported an average length of 10.62 mm, also significantly greater in men than women (11.1 mm vs. 9.7 mm) [6]. Similar trends were observed in other ethnic groups, with Jayasinghe et al. documenting a mean length of 9.91 mm in the Sri Lankan population and Ishii et al. reporting 10.3 mm in Japanese people [9,12]. These findings suggest some degree of ethnic variability but generally place the length of the incisive canal within a clinical reference range of 9.5-11.5 mm.

The mean palatal bone length in this study was comparable to those described by Soumya et al. and Panda et al., who documented means of 10.8 mm and 11.1 mm, respectively, further confirming this anatomical similarity in South Asian populations [6,8]. This study also showed a marked gender difference, with males exhibiting a significantly greater mean palatal bone compared to females in the Pakistani subpopulation. This study reports an average palatal bone thickness of 10.80±2.21 mm, which was significantly greater in males (11.96 mm) than in females (9.80 mm, p<0.05). Similar findings reported a thicker palatal bone in men compared to women, suggesting a biochemical advantage in implant stability [13-15].

The width of the anterior bone to the canal averaged 6.43±1.59 mm. Where there were no significant gender differences, age had a notable impact. Individuals aged 61-70 years had significantly reduced mean anterior bone width (4.85 mm) compared to those aged 10-20 years (6.94 mm), indicating an age-associated decline (p=0.010). Similar trends are echoed in the work of Mardinger et al., who highlighted the effects of aging and bone resorption on anterior maxillary dimensions, often limiting implant placement options [16]. The trend also aligns with various research findings by reported anterior bone width narrowing below 5 mm in older patients, further reinforcing the clinical importance of age-related variation [3,6,17].

The mean posterior bone width was greater in males than in females. These measurements are supported by findings that show males have larger dimensions in both the incisive canal and the surrounding bone structures [6,18]. Aging was associated with a reduction in posterior bone width, leading to thinning of bone in the region. They also highlighted the surgical risk of canal perforation in cases of diminished posterior bone [17].

In terms of dentition, the incisor root length of the incisor was significantly greater in males compared to females (p<0.05), whereas root width at the CEJ showed no gender difference. These results are close to those reported by others who documented the same findings [2,19]. Given the proximity of the incisive canal to the apices of maxillary central incisors, particularly in older patients with alveolar bone loss, this is vital for implant surgeries in the anterior part of the maxilla.

The mean mesiodistal diameter and labiopalatal diameter of the incisive foramen had slightly larger but insignificant measurements in males. These measurements are in close agreement with those documented by Soumya et al. and Jayasinghe et al., and they also affirm that individualized imaging remains essential due to interpatient variability [6,9].

The distance from incisive foramen to alveolar crest was significantly greater in males than in females. This study’s findings are consistent with those of Wahyuni et al., who reported a mean of 6.8 mm and emphasized the significance of this measurement for vertical implant placement [3]. He et al. suggested a minimum of 8 mm clearance during surgical drilling to avoid injury to neuromuscular structures in this region [4].

In this study on the Pakistani subpopulation, the most prevalent canal shape was hourglass-shaped, and the least common was cylindrical. There was no significant association between canal shape and age or gender. These observations are in accordance with the findings of Etoz and Sisman, who also reported it to be the most prevalent shape in their Turkish population. However, Bahşi et al. found it to be the second most prevalent shape among their sample [20,21]. However, contradictory findings were also reported, with cylindrical shape being the most common finding [6,16,22]. These differences may be due to racial changes and affect implant direction and length, particularly when bone thickness is diminished.

Regarding the shape of the incisive (nasopalatine) foramen, the round form was slightly more common (45%) than the oval (40%), while the heart-shaped variant was the least frequent. There were no statistically significant differences based on age or gender. These findings align with existing literature, such as Alhumaidi et al. and Sarna et al., who reported the round shape as the most prevalent, whereas Gönül et al. and Thakur et al. observed a higher frequency of oval shape of incisive foramen in their respective populations [18,23-25].

These morphometric variations in the present study, particularly influenced by age and gender, have critical implications for surgical procedures in the anterior maxilla. These include implant positioning, surgical flap planning, and local anesthetic infiltration. These findings also support the clinical value of CBCT in providing accurate preoperative assessment, as emphasized by various researchers worldwide [8,26].

Smaller bone dimensions observed in older adults and female patients, especially anterior to the canal, highlight the need for individualized treatment protocols. This study not only provides valuable quantitative reference data for clinicians working in the region, but it also reinforces patterns observed across ethical groups. By contributing specific data from the Pakistani subpopulation, this research enhances global clinical understanding and can aid practitioners worldwide when treating patients from the Pakistani ethnicity. The shape and location of the incisive canal are quite different and must be evaluated on an individual basis to avoid increased operative risk. CBCT is still a valuable diagnostic aid in contemporary implantology and oral surgery.

One limitation of this study was the relatively small sample size from a single hospital in Lahore, which may not fully capture the diversity within the Pakistani population. The cross-sectional design limits the ability to assess changes over time. Additionally, the study did not account for maxillary growth and development, as it included a wide age range without age-specific subgroup analysis. Despite these limitations, the study still offers useful insights into anatomical trends that can guide clinical practice.

## Conclusions

Males had significantly longer incisive canals, thicker palatal bone, and longer incisor roots compared to females. Anterior bone width decreased with the participants' age, especially after 60 years, which may impact implant planning. The most common shape of the incisive canal was hourglass, while the round shape was slightly more frequent than oval. No significant differences in canal or foramen shape were found across age or gender. These provide useful baseline data for the Pakistani subpopulation and can help clinicians plan surgical procedures (e.g., maxillary implant surgery) in patients with a similar background.
